# The Intratumoral Microbiota in Breast Cancer: Roles in Progression, Immunity, and Therapy

**DOI:** 10.32604/or.2026.079281

**Published:** 2026-07-16

**Authors:** Zhihao Wei, Jijie Cai, Sifen Wang, Yachen Li, Libo Luo, Jun Chen, Fuyu Li, Hongyu Nie, Ke Gong, Manbo Cai

**Affiliations:** 1 Department of Oncology, the First Affiliated Hospital, Hengyang Medical School, University of South China, Hengyang, China; 2 State Key Laboratory of Oncology in South China, Guangdong Provincial Clinical Research Center for Cancer, Sun Yat-Sen University Cancer Center, Guangzhou, China; 3 Nanchang University Queen Mary School, Nanchang, China; 4 Department of Thyroid and Breast Surgery, Changde Hospital, Xiangya School of Medicine, Central South University (The First People’s Hospital of Changde City), Changde, China

**Keywords:** Breast cancer (BC), intratumoral microbiota, tumor progression, chemotherapy resistance, immunotherapy

## Abstract

Breast cancer (BC) remains a leading cause of cancer-related mortality worldwide, and accumulating evidence suggests that tumor-associated microbiota may contribute to disease heterogeneity beyond host genetic and immune determinants. Advances in sequencing and multi-omics technologies have uncovered a reproducible intratumoral microbiome in BC, with distinct compositional patterns associated with molecular subtypes, clinicopathological features, and clinical outcomes. Alterations in specific microbial taxa have also been linked to tumor immune status, metastatic potential, and therapeutic sensitivity, underscoring their potential value in disease stratification and prognostic assessment. Although breast tissue represents a low-biomass environment, multiple studies employing stringent contamination control strategies have confirmed the reliability of these microbial signals. Experimental evidence further demonstrates that intratumoral microbes are functionally active components of the tumor microenvironment (TME), influencing tumor progression and metastasis through immune modulation, inflammatory signaling, and metabolic or hormonal reprogramming, while also shaping responses to chemotherapy and immunotherapy. This review summarizes current knowledge on the compositional features, functional mechanisms, and clinical relevance of the BC intratumoral microbiome, highlights methodological challenges in low-biomass profiling, and discusses future directions for translating these findings into clinically actionable strategies. The aim of this review is to systematically evaluate the role of the intratumoral microbiome in breast cancer pathogenesis and treatment, and to propose a framework for translating current findings into clinical practice.

## 1 Introduction

Breast cancer (BC) is the most commonly diagnosed malignancy in women worldwide and remains a leading cause of cancer-related mortality [1]. Despite substantial advances in understanding genetic, endocrine, and immune determinants of disease progression, these factors alone do not fully account for the heterogeneity observed in tumor behavior, metastatic potential, and therapeutic response [2].

Recent advances in sequencing and multi-omics technologies have challenged the long-standing assumption that tumor tissues are sterile, revealing the presence of microbial signals across multiple solid tumors, including BC [3,4]. Independent studies have reported reproducible microbial signatures within breast tumor tissues, supporting the existence of a distinct intratumoral microbiome [3,5]. Unlike high-biomass settings such as the gut in colorectal cancer patients, where microbial communities are abundant and relatively easy to characterize, breast tissue is characterized by a naturally low microbial biomass. This low-biomass feature introduces substantial technical challenges, including heightened susceptibility to contamination, difficulties in distinguishing true microbial signals from background noise, and the need for stringent experimental and computational controls. Therefore, the development and application of novel, contamination-aware detection methods are critical for advancing research in this field. For example, the 16S sequencing technology, utilizing the SMURF algorithm based on expectation-maximization, enables joint reconstruction of sequence information from five short amplification regions through iterative optimization to infer the most probable complete 16S rRNA sequence combination for each sample. This strategy maximizes the recovery of the original 16S rRNA gene information and significantly improves the accuracy of microbial species identification in low-biomass samples [6]. Emerging evidence suggests that these tumor-associated microbes may influence cancer biology through immune modulation, inflammatory signaling, metabolic reprogramming, and interactions with anticancer therapies [7,8,9]. However, interpretation of intratumoral microbiome data in BC is complicated by the low-biomass nature of breast tissue and substantial methodological heterogeneity across studies, necessitating careful synthesis of available evidence.

In this review, we summarize current knowledge on the BC intratumoral microbiome, focusing on its compositional features, heterogeneity across molecular subtypes, associations with clinicopathological characteristics and clinical outcomes, and experimentally supported functional roles in tumor progression and treatment response. We also highlight key methodological challenges and outline future directions for translating intratumoral microbiome research into clinically relevant insights for BC. This review aims to provide a concise overview of the current understanding of the intratumoral microbiome in breast cancer and its potential clinical implications.

## 2 Compositional Landscape of the Intratumoral Microbiome in BC

### 2.1 Composition of the Intratumoral Microbiome in BC

Multiple independent studies have characterized the composition of the intratumoral microbiome in BC using 16S rRNA gene sequencing [10], RNA-seq–based microbial transcript detection, microarray hybridization, and multi-region 16S rDNA amplification. Despite substantial heterogeneity in study design and analytical pipelines, a coherent and reproducible compositional pattern emerges at both the phylum and genus levels [11].

At the phylum level, breast tumor tissues are consistently dominated by *Proteobacteria*, *Firmicutes*, and *Actinobacteria*. Early studies based on aseptically collected breast tissues identified *Proteobacteria* as the most abundant phylum in samples, although relative abundances varied according to disease status [12]. Importantly, *Proteobacteria* and *Firmicutes* remained the predominant phyla in cancer tissues after stringent removal of environmental and skin-associated contaminants, confirming that their presence was not solely attributable to contamination [4]. RNA-seq–based analyses provided complementary evidence by detecting transcriptionally active bacteria within breast tissues. Analyses of tumor and normal breast tissue transcriptomes from geographically distinct cohorts demonstrated that *Proteobacteria* accounted for approximately 42–47% of all bacterial transcripts, followed by *Firmicutes* and *Actinobacteria* [13], indicating that *Proteobacteria* are not only present but also metabolically active within the breast tumor microenvironment (TME).

Comparative analyses of paired tumor and adjacent normal tissues revealed systematic phylum-level shifts associated with malignancy. Esposito et al. reported that tumor tissues exhibited relative enrichment of *Firmicutes* and *Alphaproteobacteria*, whereas non-tumoral paired tissues were significantly enriched in *Actinobacteria* [14]. Population-based studies further corroborated these trends. Smith et al. reported a reduction of *Actinobacteria* and an increase in *Firmicutes*-related taxa in tumor tissues compared with normal and tumor-adjacent breast tissues [15]. Similarly, Tzeng et al. observed significantly reduced alpha diversity in tumor tissues accompanied by a shift toward *Proteobacteria*- and *Firmicutes*-dominated microbial communities, whereas healthy and high-risk breast tissues retained higher proportions of *Actinobacteria* [16]. Such discrepancies are likely attributable to methodological differences, including detection of intracellular bacteria and variations in normalization strategies, rather than fundamentally contradictory biological findings. Collectively, these studies indicate that *Proteobacteria*, *Firmicutes*, and *Actinobacteria* constitute the core phyla of the breast intratumoral microbiome, with tumor development most commonly associated with increased relative abundance of *Proteobacteria* and *Firmicutes* and a concomitant reduction of *Actinobacteria*.

### 2.2 Genus-Level Insights and Tumor-Specific Signatures

At the genus level, the intratumoral microbiome exhibits more pronounced tumor-associated signatures, although inter-study variability is greater than that observed at the phylum level. Among *Proteobacteria*-associated genera, *Pseudomonas* is the most consistently reported tumor-enriched genus. In a paired-sample analysis, Esposito et al. identified *Pseudomonas* as one of the three most abundant genera in tumor cores, accounting for approximately 15% of total bacterial abundance and showing significant enrichment relative to matched non-tumoral tissues [14]. Urbaniak et al. independently reported increased *Pseudomonas* abundance in malignant breast tissues following rigorous contaminant filtering [12], and population-level analyses further supported this association, with *Pseudomonas* enriched in tumor and tumor-adjacent tissues compared with normal breast tissues [15]. *Acinetobacter* is another frequently reported tumor-associated genus. Esposito et al. identified *Acinetobacter* as a dominant genus in tumor tissues, representing more than 15% of the bacterial community [14], and earlier breast tissue microbiome studies similarly reported *Acinetobacter* among the most abundant genera in cancer samples. Members of the *Enterobacteriaceae* family also feature prominently at the genus level. Urbaniak et al. observed increased abundance of unclassified *Enterobacteriaceae* in BC patients compared with healthy controls [4], while Tzeng et al. further identified genera such as *Proteus* and *Escherichia*/*Shigella* as characteristic of tumor tissues but largely absent from healthy breast tissues [16].

Additional *Proteobacteria*-associated genera reported in breast tumors include *Ralstonia*, *Sphingomonas*, *Caulobacter*, *Bradyrhizobium*, and *Burkholderia*, although the directionality of their association varies among studies. For example, *Sphingomonas* was enriched in pre-diagnostic breast tissues in women who later developed BC [17], whereas other studies reported higher abundance in tumor-adjacent rather than tumor tissues [15]. These discrepancies may stem from differences in cohort geography, such as Midwest versus Southeast U.S. populations, tissue sampling protocols, such as truly healthy tissue banks versus surgical tissue networks, and the specific disease stage being examined, such as pre-diagnostic versus established cancer.

*Firmicutes*-associated genera show more heterogeneous patterns. *Staphylococcus* is among the most frequently detected genera across breast tissue microbiome studies, with some analyses reporting enrichment in tumor tissues [18,19], while others observed higher abundance in healthy or tumor-adjacent tissues [16]. *Bacillus* was reported as enriched in malignant tissues relative to healthy controls [4]. Other *Firmicutes* genera, including *Enterococcus*, *Lactococcus*, and *Clostridium*, were reported as increased in tumor tissues in selected cohorts [15,17], although these findings were not universal.

In contrast, several *Actinobacteria*-associated genera are consistently depleted in tumor tissues. *Propionibacterium* represents the most robustly reported example. Paired-sample analyses demonstrated significant enrichment of *Propionibacterium* species in non-tumoral tissues compared with tumor cores [14], and transcriptomic studies confirmed higher abundance of *Propionibacterium*-derived transcripts in healthy breast tissues [13]. Large cohort analyses further demonstrated marked depletion of this genus in tumor tissues [16]. *Corynebacterium* exhibits a similar distribution pattern, with higher abundance in healthy breast tissues and reduced abundance following tumor development [17]. Additional *Actinobacteria* genera, including *Micrococcus*, *Bifidobacterium*, *Collinsella*, and *Adlercreutzia*, were also reported as enriched in healthy tissues but depleted in tumor tissues, despite their relatively low abundance [13]. Finally, some genera appear to be associated with specific BC subtypes or clinical contexts. *Sphingobacterium* was reported as enriched in tumor tissues and subsequently validated as a tumor-resident genus in triple-negative breast cancer (TNBC) [18], while microarray-based oncobiome studies further identified subtype-associated enrichment of genera such as *Aggregatibacter*, *Aeromonas*, *Alcaligenes*, *Capnocytophaga*, and *Pasteurella* across receptor-defined BC subtypes [20].

### 2.3 Discussion and Perspectives

These findings underscore the potential of the intratumoral microbiome as a diagnostic and prognostic tool. However, they also raise critical questions about the functional role of the microbiome in BC development and progression. While the intratumoral microbiome appears to be shaped predominantly by the local tissue TME, rather than by passive translocation from systemic sources, further research is needed. Integrating the profiling of tumor, adjacent tissue, and blood microbiomes could provide a more comprehensive understanding of the complex interactions between these distinct microbial communities and their collective influence on tumorigenesis.

For a comprehensive summary of key studies characterizing the intratumoral microbiome in breast cancer, including sample types, detection methods, and enriched taxa, see Supplementary Table S1. Despite the increasing number of descriptive studies, several key challenges remain. Despite the increasing number of descriptive studies, several key challenges remain. First, most current evidence is largely associative, and the causal relationships between specific microbial taxa and tumor progression have not been fully established. Second, the low-biomass nature of breast tissue introduces substantial technical limitations, including contamination bias and variability in detection sensitivity across studies. In addition, inter-study heterogeneity in sampling strategies, sequencing platforms, and analytical pipelines further complicates cross-cohort comparisons and the identification of consistent microbial signatures.

**Figure 1:**
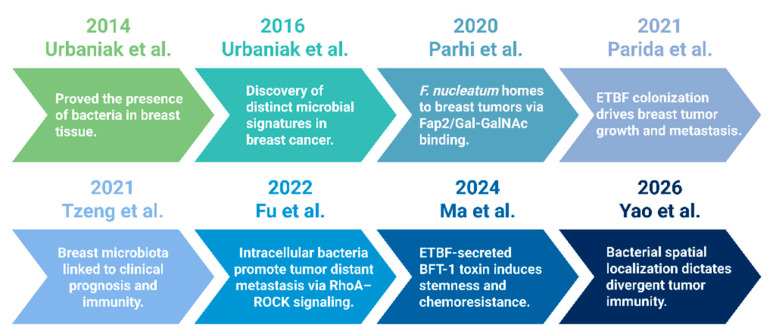
**Timeline of key milestones in breast cancer (BC) intratumoral microbiota research.** Early studies confirmed the presence of bacteria and their distinct features; subsequent research revealed mechanisms including targeted colonization of *Fusobacterium nucleatum*, Enterotoxigenic *Bacteroides fragilis* (ETBF)-driven metastasis, associations between microbiota and clinical prognosis, and RhoA-ROCK pathway-mediated distant metastasis; recent findings further elucidated ETBF-induced chemoresistance and the spatial regulation of tumor immunity by bacteria.

Key milestones from 2014 to 2026 highlight the evolution of this field—from the initial discovery of bacteria in breast tissue to the identification of molecular mechanisms that govern tumor colonization, metastasis, and immune modulation (Fig. 1). These advancements reflect a deeper understanding of the role the microbiome plays in BC pathogenesis and offer promising avenues for future research and clinical applications.

Looking forward, future studies should prioritize standardized methodologies, including contamination-aware protocols and unified bioinformatic pipelines, to improve reproducibility. The integration of multi-omics approaches, such as metagenomics, transcriptomics, and spatial profiling, will be essential to elucidate the functional roles of tumor-associated microbiota. Moreover, longitudinal and mechanistic studies are required to distinguish causal microbial drivers from correlative patterns. Ultimately, translating these findings into clinical applications will depend on the identification of robust microbial biomarkers and the development of microbiome-targeted interventions, which may contribute to improved diagnosis, prognosis, and personalized therapeutic strategies in breast cancer.

## 3 Intratumoral Microbial Heterogeneity across BC Molecular Subtypes

Multiple investigations have reported that intratumoral bacterial composition differs across BC molecular subtypes. Across independent cohorts, microbial profiles were shown to vary among estrogen receptor–positive, hormone receptor–positive, HER2-positive, and TNBCs, although the specific taxa contributing to subtype separation were not consistent across studies [15,20]. Additional support for subtype-associated variation has come from analyses capturing bacterial transcriptional signatures within tumor tissue, suggesting that subtype differences may reflect variation in bacterial activity as well as bacterial presence [13].

A consistent observation across subtype-focused studies is that TNBC exhibits the lowest intratumoral microbial complexity. TNBC samples were reported to contain fewer detectable microbial signatures and to form distinct clusters in multivariate analyses, separating them from receptor-positive tumors [20]. Although certain taxa were described as relatively enriched in TNBC compared with other subtypes, the identities of these taxa differed across cohorts, supporting a general conclusion of subtype-linked compositional differences rather than a uniform TNBC-specific microbial signature.

In contrast, estrogen receptor–positive tumors were repeatedly reported to harbor more complex intratumoral microbial profiles than TNBC. These tumors showed broader detectable bacterial signals, with microbial communities largely dominated by *Proteobacteria* but also including *Firmicutes*- and *Actinobacteria*-associated lineages [16,20]. In tissue-based analyses evaluating multiple prognostic features simultaneously, estrogen receptor status was among the tumor characteristics associated with differential abundance of multiple genera, reinforcing the association between receptor status and microbiome variation within tumors [16].

HER2-positive and hormone receptor–positive (HR^+^) tumors were generally reported to exhibit intermediate microbial diversity. Subtype-stratified analyses identified subtype-associated microbial biomarkers across luminal A, luminal B, HER2, and TNBC tumors, although the taxa highlighted differed depending on cohort composition and analytical thresholds [15]. Collectively, these findings indicate that subtype-associated microbial variation is detectable but heterogeneous and likely influenced by both biological differences and cohort-specific factors.

## 4 Associations between the Intratumoral Microbiome and Clinicopathological Features

Associations between intratumoral bacterial composition and clinicopathological features have been described in several cohorts. In a large tissue-based study controlling for key confounders, multiple genera were found to correlate with tumor stage, and some taxa were detected exclusively in higher-stage tumors, suggesting stage-related variation in microbial composition, though such findings remain correlative. Genera such as *Porphyromonas* and *Fusobacterium* showed higher relative abundances in higher-stage tumors in some datasets, illustrating that certain taxa may track with disease progression in specific datasets [16]. However, these patterns were not uniformly observed across studies, indicating that stage-associated microbial differences are cohort-dependent. Importantly, given the descriptive and cross-sectional nature of these studies, all such findings should be interpreted as associative rather than causal. Whether these microbial differences contribute to disease progression or merely reflect the evolving TME with advancing stage remains to be determined and requires mechanistic investigation.

Microbiome differences have also been reported in relation to disease context outside the tumor core. Comparisons between normal adjacent tissue from cancer patients and tissue from healthy or benign conditions revealed distinct community profiles and differential abundance of multiple genera, further supporting the concept that clinically defined tissue context correlates with measurable microbiome variation even in non-tumoral breast tissue [4,21]. As with the findings above, these observations reflect correlative patterns and do not imply causation.

Histological grade has similarly been linked to microbiome variation. Studies stratifying malignant tissue by grade reported differences in the relative abundance of selected families and genera across grade categories, along with grade-dependent variation in predicted microbial functional pathways [19]. In a larger cohort evaluating multiple prognostic variables, grade was again associated with differential microbial abundance patterns, supporting a relationship between tumor differentiation status and intratumoral microbiome composition in at least some datasets [16].

Metastatic features have also been examined. Node-positive tumors were associated with distinct microbial abundance patterns compared with node-negative tumors in tissue cohort analyses [16]. In parallel, transcriptome-based microbial profiling reported differences in bacterial transcriptional patterns between tumors from patients with and without circulating tumor cells, suggesting that microbial activity signatures may vary along metastatic-related clinical axes [13].

## 5 Clinical Outcome and Treatment-Associated Microbial Signatures

A study reported relationships between intratumoral microbial patterns and clinical outcomes, particularly in the context of TNBC and treatment response. In TNBC, tumors clustered by the intensity of selected microbial signatures showed differences in disease-free interval, with the higher-signal cluster exhibiting smaller tumor size and longer disease-free intervals within the available follow-up data. The finding demonstrates that intratumoral microbial signature patterns can correlate with clinically relevant heterogeneity within a molecular subtype, without implying causation [20].

Intratumoral microbial composition has also been reported to differ according to treatment response. In a cohort comparing neoadjuvant chemotherapy responders and non-responders, tumors from non-responders exhibited higher bacterial diversity and differences in class-level composition compared with complete responders, suggesting that tumor-resident microbial patterns may vary with therapeutic response status. Although limited by sample size, this study provides direct evidence that response-associated microbial differences can be detected in breast tumor tissue [22].

Finally, broader clinical relevance is supported by studies linking microbial composition to prognostic features even when survival endpoints were not directly modeled. Associations between microbial taxa and tumor stage, grade, receptor status, lymphovascular invasion, or node status have been reported in tissue cohorts [16]. In addition, microbial transcriptional signatures have been described along clinically relevant axes such as circulating tumor cell status and receptor-defined phenotypes [13]. Together, these findings indicate that intratumoral microbial profiling can capture variation aligned with prognosis and treatment response, while the specific taxa and effect directions vary across cohorts.

## 6 Functional Roles of Tumor-Associated Microbiota in BC Progression and Metastasis

### 6.1 Functional Roles of Enterotoxigenic Bacteroides fragilis (ETBF) and Fusobacterium nucleatum in BC Progression

#### 6.1.1 Origins of Intratumoral Microbiota and Tumor Colonization

The origins of breast intratumoral microbiota are thought to involve the enteromammary axis, wherein gut-derived microbes reach breast tissue via hematogenous dissemination. Supporting this, Parhi et al. demonstrated that intravenously administered *Fusobacterium nucleatum* selectively colonizes mammary tumors in a Fap2–Gal-GalNAc-dependent manner, with bacterial abundance in human BC samples correlating with Gal-GalNAc expression levels [23]. These findings provide a mechanistic link between bacterial dissemination from distant sites and tumor-specific colonization, logically connecting microbial origins with their functional impact on tumor progression. An increasing body of evidence indicates that tumor-associated microbiota are not merely passive bystanders but functionally active components of the BC microenvironment, exerting profound effects on tumor initiation, progression, metastasis, and therapeutic response [24,25].

#### 6.1.2 Oncogenic Mechanisms of ETBF in Breast Cancer Progression

Early investigations revealed that ETBF, a toxin-producing anaerobe traditionally associated with colorectal carcinogenesis, is also present in breast tissue and exhibits strong oncogenic potential. ETBF colonization of the mammary gland or exposure to its secreted toxin BFT induces epithelial hyperplasia and promotes breast tumor growth and metastatic dissemination through sustained activation of the Notch1 and β-catenin signaling pathways [26]. Building on these findings, subsequent studies demonstrated that ETBF-secreted BFT-1 directly binds to and stabilizes NOD1 in BC stem cells, leading to phosphorylation-dependent degradation of NUMB, activation of the NOTCH1–HEY1 axis, and enhanced resistance to taxane-based chemotherapy [22].

Beyond ETBF, recent studies have highlighted the broader contribution of intratumoral and intracellular microbiota to BC metastasis. Using spontaneous BC mouse models, tumor-resident intracellular bacteria were shown to promote metastatic colonization by enhancing tumor cell survival during circulation, particularly by increasing resistance to fluid shear stress through actin cytoskeleton reorganization [3]. These findings suggest a previously underappreciated role of microbiota in late-stage metastatic processes.

#### 6.1.3 Roles of Fusobacterium nucleatum in Breast Cancer Progression

Among the tumor-associated bacteria identified in BC, *Fusobacterium nucleatum* has emerged as one of the most extensively studied species. Clinical and experimental evidence demonstrates that *Fusobacterium nucleatum* preferentially colonizes breast tumors in a Fap2-dependent manner by binding tumor-expressed Gal-GalNAc, with bacterial abundance correlating with tumor progression and metastatic burden [23]. Functionally, *Fusobacterium nucleatum* suppresses antitumor immune responses by reducing tumor-infiltrating T cells, thereby facilitating tumor growth and distant metastasis. Complementary mechanistic studies further revealed that intratumoral *Fusobacterium nucleatum* induces IL-1β secretion via NLRP3 inflammasome activation in BC cells, directly stimulating tumor cell proliferation and establishing a pro-inflammatory TME conducive to cancer progression [27].

Recent work has expanded the mechanistic landscape of *Fusobacterium nucleatum*–mediated tumor promotion. *Fusobacterium nucleatum*–derived small extracellular vesicles were shown to significantly enhance BC cell proliferation, migration, invasion, and metastatic potential through activation of TLR4 signaling pathways, both *in vitro* and *in vivo* [28]. At the post-transcriptional level, *Fusobacterium nucleatum* infection promotes epithelial–mesenchymal transition and cell migration via upregulation of miR-21-3p and subsequent suppression of the tumor suppressor FOXO3 [29]. Moreover, intracellular *Fusobacterium nucleatum* infection has been demonstrated to induce METTL3-mediated m^6^A RNA methylation, increasing the stability of oncogenic transcripts such as c-Myc and driving metastatic progression [30]. Importantly, spatial multi-omics profiling revealed marked intratumoral heterogeneity of *Fusobacterium nucleatum* distribution, with colonized tumor regions exhibiting activation of MAPK signaling and upregulation of key effectors such as VEGFD and PAK1, which are critical drivers of tumor proliferation and migration [31]. However, contrasting with some reports suggesting AMPK pathway activation by *Fusobacterium nucleatum*, Sun et al. identified AMPK inhibition in colorectal cancer through a miRNA-mediated metabolic mechanism [32]. These differences may be attributed to tumor type–specific metabolic contexts, variations in experimental models, and differences in bacterial localization or metabolite production.

#### 6.1.4 Conclusions and Perspectives

Current evidence highlights that tumor-associated microbiota, particularly ETBF and *Fusobacterium nucleatum*, play multifaceted roles in breast cancer progression and metastasis. These microbes contribute to tumor development through diverse mechanisms, including activation of oncogenic signaling pathways, modulation of host immune responses, promotion of epithelial–mesenchymal transition, and enhancement of metastatic fitness. In addition, their ability to colonize tumor tissues in a selective and spatially heterogeneous manner further underscores their active participation in shaping the tumor TME.

However, several challenges remain. Most existing studies are based on experimental models or cross-sectional analyses, and the causal relationships between specific microbial taxa and tumor progression are not yet fully established. Furthermore, the observed discrepancies in signaling pathways, such as AMPK regulation, suggest that microbial effects are highly context-dependent and influenced by tumor type, microenvironmental conditions, and methodological differences.

Future research should focus on integrating spatial multi-omics, functional validation, and longitudinal studies to better define the dynamic interactions between microbiota and tumor cells. A deeper understanding of these mechanisms may facilitate the development of microbiome-targeted therapeutic strategies and improve precision treatment approaches in breast cancer.

### 6.2 Other Tumor-Associated Bacteria and Their Roles in BC Progression

**Figure 2:**
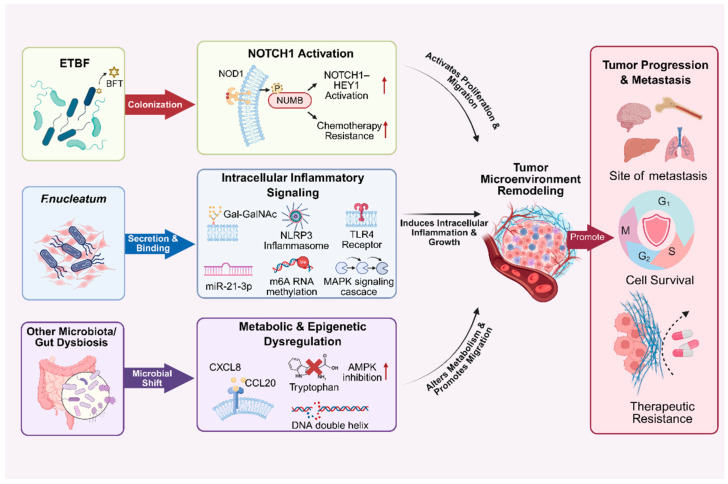
**Functional roles of tumor-associated microbiota in BC progression and metastasis.** Tumor-associated microbiota, including ETBF and *Fusobacterium nucleatum*, promote breast cancer progression through activation of oncogenic signaling pathways, induction of inflammatory responses, and metabolic dysregulation, ultimately contributing to TME remodeling, metastasis, and therapeutic resistance. Abbreviations: ETBF, enterotoxigenic *Bacteroides fragilis*; BFT, *Bacteroides fragilis* toxin; NOD1, nucleotide-binding oligomerization domain-containing protein 1; NUMB, NUMB endocytic adaptor protein; NOTCH1, notch receptor 1; HEY1, hes-related family bHLH transcription factor with YRPW motif 1; NLRP3, NLR family pyrin domain containing 3; TLR4, toll-like receptor 4; CXCL8, C–X–C motif chemokine ligand 8; CCL20, C–C motif chemokine ligand 20. The figure was created using BioRender.com.

In addition to *Fusobacterium nucleatum*, other intratumoral bacteria have been implicated in BC progression through immune and metabolic mechanisms. *Sphingobacterium multivorum* was identified as a dominant intratumoral species that accelerates tumor growth and diminishes the therapeutic efficacy of anti–PD-1 immunotherapy by inducing chemokines such as CCL20 and CXCL8, leading to increased regulatory T cell infiltration and reduced CD8^+^ T cell–mediated immune surveillance [18]. Furthermore, gut microbiota dysbiosis has also been linked to BC progression. Enrichment of *Prevotella copri* in the gut microbiome of BC patients promotes tumor growth by consuming host tryptophan and depleting the endogenous antitumor metabolite indole-3-pyruvic acid, thereby strengthening UHRF1-mediated negative regulation of AMPK signaling and inducing epigenetic reprogramming in tumor cells [33]. Finally, translational studies have begun to explore therapeutic strategies targeting microbe-associated signaling pathways, as selective inhibition of Wnt signaling using a *Clostridioides difficile* toxin B fragment effectively suppresses tumor growth and chemotherapy-resistant tumor-initiating cells in BC models while sparing normal Wnt-dependent tissue [34] (Fig. 2). 

The findings outlined above suggest that the intratumoral microbiome plays a critical role in BC progression. Both ETBF and *Fusobacterium nucleatum* have demonstrated significant contributions to tumor initiation, progression, and metastasis through immune modulation, metabolic alterations, and direct effects on tumor cells. These microbes actively influence the TME by enhancing tumor cell survival, promoting immune evasion, and driving resistance to therapies. As such, the tumor-associated microbiota should no longer be considered passive bystanders but as active participants in BC pathogenesis.

### 6.3 Limitations and Future Perspectives

Although accumulating evidence supports critical roles for tumor-associated microbiota, particularly ETBF and *Fusobacterium nucleatum*, in breast cancer progression, several limitations remain. Most current findings are derived from experimental models, and the causal relationships between specific microbes and tumor development are not yet fully established. In addition, the effects of these bacteria appear to be highly context-dependent, as reflected by discrepancies in reported signaling pathways, which may be influenced by tumor type, microenvironmental conditions, and methodological differences.

Moreover, the complex interactions between intratumoral microbiota and the tumor microenvironment, including immune regulation and metastatic processes, are not yet fully understood. Current studies often focus on individual mechanisms, whereas the integrated effects of microbial colonization, spatial distribution, and host responses require further investigation. Future research should aim to combine multi-omics approaches with functional validation to better define the roles of tumor-associated microbiota in breast cancer. Clarifying these mechanisms may facilitate the development of microbiome-targeted therapeutic strategies and improve precision treatment approaches. 

## 7 Intratumoral Bacteria as Modulators of the Tumor Immune Microenvironment

### 7.1 Intratumoral Bacteria Modulate Immune Cell Infiltration and Promote Immune Evasion

In the BC immune microenvironment, intratumoral bacteria profoundly affect immune cell infiltration, and immune escape or immunogenic cell death via multiple mechanisms. Instead, microbes in the TME of BC actively regulate the entry of immune cells. They can be recognized by pattern recognition receptors, especially Toll-like receptors (TLRs), using pathogen-associated molecular patterns (PAMPs) such as lipopolysaccharide (LPS), lipoteichoic acid (LTA), or flagellin, which are present on microbes. *Fusobacterium nucleatum* interacts with Gal-GalNAc on the surface of BC cells via its Fap2 adhesin, facilitating bacterial entry into tumor tissue. Correlative evidence from clinical cohorts has shown that higher *Fusobacterium nucleatum* abundance is associated with reduced T cell infiltration and accelerated tumor progression [35]. Causal evidence from mouse models further supports these associations, demonstrating that *Fusobacterium nucleatum* colonization directly inhibits cytotoxic T lymphocyte infiltration and promotes an immunosuppressive TME. Moreover, LPS-rich bacteria can enhance the infiltration of tumor-associated macrophages and myeloid-derived suppressor cells (MDSCs) by upregulating TLR4 and activating its downstream signaling pathways. These immune cells evade immune surveillance through immunosuppressive mechanisms [36,37].

### 7.2 Intratumoral Bacteria Regulate Immune through Cytokine Signaling and Immune Checkpoint Pathways

Intratumoral bacteria not only facilitate immune evasion by modulating immune cell infiltration but also increase tumor cell immune tolerance through mechanisms such as cytokine modulation and the production of immune checkpoint proteins [38,39]. Correlational analyses have revealed that *Fusobacterium nucleatum* abundance positively correlates with PD-L1 expression on tumor cells in BC patient samples, and this increased expression is associated with reduced CD8^+^ T cell function [40]. Furthermore, as shown by Parhi et al., a high amount of *Fusobacterium nucleatum* DNA is found within BC cells. The presence not only facilitates the development and metastasis of BC but also weakens anti-tumor immunity. It achieves this by activating the immune-suppressive checkpoint receptors TIGIT and CEACAM1, which ultimately leads to a reduction in the numbers of CD4^+^ and CD8^+^ T cells [23]. 

Moreover, bacterial metabolites may promote antitumor immunity by inducing immunogenic cell death. TMAO, for instance, a substance produced by the bacterial genus Clostridiales, has been shown to activate PERK kinase in the endoplasmic reticulum of TNBC cells. The subsequent induction of pyroptosis contributes to enhanced CD8^+^ T cell-mediated antitumor immune responses [41]. However, microbial-derived signals can also exert pro-tumorigenic effects under certain conditions, highlighting the complex and context-dependent roles of microbiota in shaping tumor immunity and progression [41,42].

Intratumoral bacteria activate downstream NF-κB and MAPK signaling pathways by interacting with TLR receptors and PAMPs, thereby promoting the release of pro-inflammatory cytokines such as IL-6, TNF-α, and IL-1β. Notably, it has been well established that bacterial components trigger NF-κB signaling, and this induced pro-inflammatory axis has been implicated in the pathogenesis and immunosuppressive TME [43]. These cytokines are crucial in maintaining the chronic inflammatory state of the tumor immune microenvironment. Specifically, IL-6 not only serves as a key marker of inflammation but also plays a vital role in tumor cell proliferation, angiogenesis, and immune evasion. LPS-rich bacteria activate the LPS/S100A7/TLR4/RAGE axis via TLR4, triggering IL-6 release and accelerating tumor growth and metastasis [37,44]. Moreover, bacterial flagellin can stimulate TLR5, triggering IL-6 secretion and driving malignant progression [45]. Collectively, these findings highlight that intratumoral bacteria play multifaceted roles in shaping the breast cancer immune microenvironment. By modulating immune cell infiltration, regulating cytokine networks, and activating immune checkpoint pathways, tumor-associated microbiota contribute to immune evasion and tumor progression. The interplay between microbial signals and host immune responses not only sustains a chronic inflammatory and immunosuppressive milieu but also influences therapeutic outcomes. Therefore, a deeper understanding of microbiota–immune interactions may provide novel opportunities for improving immunotherapeutic strategies and developing microbiome-targeted interventions in BC.

## 8 Metabolic and Hormonal Reprogramming Driven by the Intratumoral Microbiome

In addition to the well-known inflammatory and immunomodulatory pathways, the microbiota in BC also contributes to metabolic and hormonal reprogramming [46,47]. Take Bacillus cereus as an instance. It activates 5α-steroid hydroxylase, an enzyme that can break down steroid hormones such as progesterone and testosterone, thereby exerting pro-carcinogenic effects. This enzymatic action leads to the production of 5α-pregnane-3,20-dione (5αP). *In vitro* studies have demonstrated that 5αP can boost the proliferation of BC cells [48,49,50].

Research has also shown that when examining the microbial populations in the nipple aspirate fluid of healthy individuals and BC survivors, distinct differences are evident. Specifically, the incidence of Alistipes is higher in BC samples. Moreover, elevated levels of β-glucuronidase in BC tissues can reverse the conjugation of glucuronidated estrogen. As a result, active estrogen is released, which in turn promotes the progression of BC [51,52].

Regarding lipid metabolism, tissues adjacent to tumors exhibit elevated levels of lysophosphatidylcholines (LysoPCs) and oxidized cholesteryl esters (oxCEs). At the same time, the levels of diacylglycerols (DGs), phosphatidylethanolamines (PEs), and ceramides (Cer) are decreased. These lipid changes may be associated with the presence of certain commensal bacteria [53].

Some bacteria also affect BC through their metabolites. For example, the secretome of *E. coli* can disrupt the metabolic pathways of BC cells. Clostridium species can produce deoxycholic acid, which promotes BC cell proliferation. The exopolysaccharides of B. subtilis can enhance the invasiveness of BC cells. *Fusobacterium nucleatum* can produce metabolic by-products, such as short-chain fatty acids, which can influence the TME and the metabolism of cancer cells, thereby affecting the growth and survival of cancer cells [54].

Notably, Xuan et al. found that *Sphingomonas yanoikuyae* is prevalent in normal tissues and has protective functions against BC. This bacterium expresses ligands that activate invariant NKT (iNKT) cells, which provide a defence against cancer cells. Moreover, S. yanoikuyae can modulate estrogen metabolism and trigger TLR5-dependent pathways, both of which are involved in inhibiting BC development [55]. Moreover, *Streptococcus* can produce antioxidant metabolites. These metabolites can neutralize peroxide and superoxide radicals, thereby preventing DNA damage [56].

The recognition of the intratumoral microbiome as a key regulator of tumor biology provides a novel conceptual framework for treatment. Insights into its immunosuppressive and metabolically active roles directly inform strategic approaches to augment standard therapies. Targeting these microbial functions thus represents an actionable avenue to reprogram the TME and enhance responses to immune checkpoint blockade and classical chemotherapeutics.

## 9 Intratumoral Microbiota and Immunotherapy Response

### 9.1 Intratumoral Microbiota Shapes Immune Checkpoint Inhibitor Responses

Immune checkpoint inhibitor (ICI) therapy has demonstrated remarkable efficacy in treating various malignancies, especially in advanced-stage patients. The key to ICI therapy lies in activating the anti-tumor activity of cytotoxic T cells within the TME and reducing their dysfunction or exhaustion. However, TNBC generally exhibits limited T-cell infiltration and a low tumor mutational burden, leading to poor responsiveness to ICIs. Notably, recent studies have revealed that the body’s commensal microbiota is closely associated with anti-cancer immune responses, which provides a potential breakthrough for improving the efficacy of ICI therapy in TNBC. The intratumoral microbiota influences anti-cancer immune responses within the TME through multiple mechanisms: it can stimulate T-cell responses to microbial antigens localized in the tumor, mediate pro-immune or anti-inflammatory effects via pattern-recognition receptors on immune cells in the TME, and regulate the local immune status of the host through bacterial metabolites secreted directly into the tumor niche. Importantly, the composition of the intratumoral microbiota is tightly linked to the response to immunotherapy. Non-responders to ICIs often exhibit dysregulated intratumoral microbiota, which may drive abnormal local inflammatory responses in the TME and alter T-cell differentiation within the tumor. For instance, specific intratumoral bacterial strains can enhance the anti-tumor effect of ICIs by promoting T-cell infiltration into the tumor. In contrast, a high abundance of certain intratumoral bacteria (e.g., some *Bacteroides* strains) is associated with elevated levels of regulatory T cells (Treg) and myeloid-derived suppressor cells (MDSC) within the TME [57]. Since Treg and MDSC are key immunosuppressive cells that locally hinder anti-tumor immune responses in the tumor, this dysregulated intratumoral microbiota may be a critical factor in the poor response to immunotherapy.

### 9.2 Intratumoral Microbiota Reprograms the Tumor Immune Microenvironment

**Figure 3:**
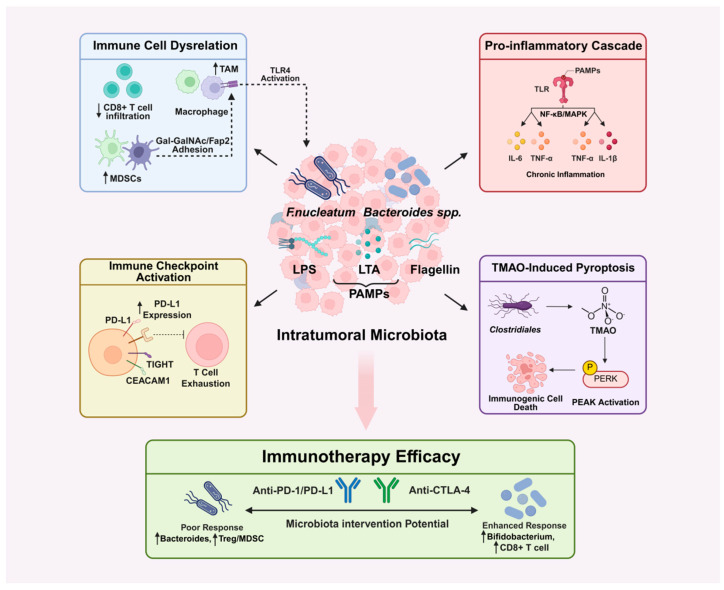
**Intratumoral Microbiota and Immunotherapy Response.** Intratumoral microbiota modulate immunotherapy efficacy in breast cancer through multiple mechanisms, including disruption of immune cell infiltration, activation of immune checkpoint pathways, induction of pro-inflammatory signaling (e.g., NF-κB/MAPK), and metabolic regulation such as TMAO-mediated pyroptosis. Key bacterial taxa, including *Fusobacterium nucleatum* and *Bacteroides* spp., interact with the TME via PAMP–TLR signaling and immune suppression, ultimately shaping responses to immune checkpoint blockade. Abbreviations: TAM, tumor-associated macrophage; CD, cluster of differentiation; MDSC, myeloid-derived suppressor cell; NF-κB, nuclear factor kappa B; MAPK, mitogen-activated protein kinase; IL, interleukin; TNF, tumor necrosis factor; PD-L1, programmed death-ligand 1; TIGIT, T cell immunoreceptor with Ig and ITIM domains; LPS, lipopolysaccharide; LTA, lipoteichoic acid; TMAO, trimethylamine N-oxide; PERK, protein kinase R-like endoplasmic reticulum kinase; CTLA, cytotoxic T-lymphocyte–associated antigen. The figure was created using BioRender.com.

Beyond the systemic commensal microbiota, the intratumoral microbiota, which closely interacts with the TME, plays a crucial role in modulating local anti-tumor immune responses. Compared to systemic microbiota, the intratumoral microbiota resides in the TME and can directly regulate the function of immune effector cells therein, either enhancing or reducing tumour immunogenicity to promote or inhibit anti-tumour immunity [58]. For BC, microbial reprogramming of the TME has shown promising therapeutic potential. For example, the previously mentioned trimethylamine N-oxide (TMAO) and its precursors have been identified as targets associated with immunotherapy resistance [41]. Elucidating the role of intratumoral microbiota as immune-sensitizing agents may thus transform immunotherapeutic strategies for BC, particularly for TNBC. As summarized in Fig. 3, the intratumoral microbiota orchestrates a complex interplay with the tumor immune microenvironment. Specific taxa, including *Fusobacterium nucleatum* and *Bacteroides* spp., contribute to immune dysregulation through multiple pathways: activation of pro-inflammatory cascades via Toll-like receptor signaling, induction of immune checkpoint molecules such as PD-L1, and recruitment of immunosuppressive cell populations including tumor-associated macrophages and MDSCs. Collectively, these microbiota-driven alterations shape the efficacy of immune checkpoint inhibitor therapy (Fig. 2).

### 9.3 Engineered Bacteria and Microbiome-Based Therapeutic Strategies

#### 9.3.1 Natural Intratumoral Microbiota and Immune Modulation

While the endogenous intratumoral microbiota naturally modulates immune responses, its clinical exploitation is limited by inherent variability and challenges in precise control. In contrast, engineered bacteria represent a distinct therapeutic strategy that leverages synthetic biology for active and precise modulation of the tumor immune microenvironment. Emerging studies suggest that the intratumoral microbiota may interact with other components of the TME, including extracellular vesicles and cytokines. Bacteria-derived extracellular vesicles can carry immunomodulatory molecules to modulate immune cell function in tumors, while intratumoral microbiota can regulate cytokine production and release. Although native microbiota exerts significant regulatory effects on immunotherapy responses, its clinical application is limited by factors such as unstable regulatory effects and difficulty in precise targeting.

#### 9.3.2 Engineered Bacteria and Synthetic Biology-Based Strategies

In contrast, engineered bacteria have emerged as promising therapeutic agents that can actively and precisely reshape the tumor immune TME to enhance treatment efficacy. Zhang et al. developed a tumor-specific bacterial vaccine (cBEV) by coating extracellular vesicles derived from BF with manganese dioxide [59]. Once internalized by dendritic cells, the manganese oxide-based vesicles activate these cells, boosting the immune response and inhibiting bacterial growth within tumors [59]. However, the clinical translation of engineered bacteria faces substantial safety and regulatory challenges, including concerns regarding bacterial persistence, potential off-target colonization, and the risk of unintended immune responses. Rigorous preclinical safety assessments and well-defined regulatory frameworks will be essential to advance these approaches toward clinical application. When combined with anti-PD-L1 antibody therapy in BC, this approach shows promising preventive and synergistic effects, demonstrating the potential of tumour-specific bacteria and their extracellular vesicles to enhance immunotherapy efficacy, particularly in TNBC. Moreover, the genetic diversity of the intra-tumoral microbiota may matter, as different strains could have distinct immunomodulatory capabilities. Analyzing its genetic makeup could identify specific strains or markers associated with better immunotherapy outcomes, enabling personalized microbiota-based immunotherapies. Overall, understanding the role of the intra-tumoral microbiota in BC immunotherapy can provide new insights into improving treatment strategies, and the impact of intratumoral microbes on the host TME’s immune status clearly deserves further in-depth exploration.

#### 9.3.3 Biomaterial Integration and Bacteria-Based Delivery Systems

Building on these natural microbiota-immune interactions, engineered bacteria have emerged as promising tools to enhance the efficacy of cancer immunotherapy. Moreover, recent advances in nanomedicine have further expanded the potential of integrating nanomaterials with bacterial platforms to modulate tumor progression, immune responses, and therapeutic outcomes [60]. For example, engineered *E. coli* MG1655 cells coated with lanthanide upconversion nanoparticles (UCNPs) were observed to accumulate at hypoxic tumor sites due to their innate chemotactic behavior [61]. This accumulation facilitated localized near-infrared (NIR) laser irradiation, which triggered the UCNPs to convert NIR light to blue light, subsequently activating the bacteria to secrete HlyE perforin and effectively targeting cancer cells with minimal side effects [61]. 

Han et al. pioneered a novel vaccination strategy utilizing *Rhodopseudomonas palustris* as a multifunctional platform. Their approach strategically relocates antigen presentation from the immunosuppressive tumor core to physiologically functional peripheral zones, thereby circumventing the TME’s inhibitory effects on immune activation. The photosynthetic bacteria serve dual roles: acting as effective photothermal agents while simultaneously functioning as antigen carriers. Through membrane engineering with maleimide groups, the modified bacteria selectively capture tumor-derived protein antigens via Michael addition chemistry and transport these antigens (with preserved immunological competence) to peritumoral antigen-presenting cells [62]. Additionally, bacterial outer membrane vesicles facilitate rapid antigen trafficking to tumor-draining lymph nodes through lymphatic channels. This innovative design enhances dendritic cell infiltration in peritumoral regions and promotes T cell activation within lymphoid tissues, ultimately strengthening systemic anti-tumor immunity while enabling concurrent photothermal ablation [62]. Despite its innovative design, the clinical application of this strategy will require careful evaluation of safety profiles, potential immunogenicity of the engineered bacterial platform, and adherence to regulatory standards for genetically modified microorganisms.

Beyond these bacterial engineering approaches, bacterial outer membrane vesicles (OMVs) have been engineered to deliver functional genetic circuits directly into tumor cells. These vesicles can transport CRISPR-based systems that reprogram cancer cells to produce immunostimulatory factors, including CXCL9 and IL12, converting the suppressive TME into an immunologically active site. In the TNBC mouse model, OMV-C9I12 treatment demonstrated significant therapeutic efficacy. When combined with anti-PD-1 therapy, the engineered OMVs achieved substantial tumor growth inhibition and reduced tumor burden compared to control groups. The platform’s ability to reprogram the TME was evidenced by enhanced recruitment and activation of human T cells within the humanised system, demonstrating translational relevance for TNBC immunotherapy [63].

#### 9.3.4 Discussion and Future Perspectives

Looking forward, the intratumoral microbiome holds promise for developing novel therapeutic strategies. Microbiome modulation through probiotics or dietary interventions may reshape the tumor microbial community to enhance treatment response. Engineered bacteria can serve as targeted drug delivery vehicles. In addition, microbial signatures may act as predictive biomarkers for patient stratification in personalized immunotherapy. Future research should focus on validating these approaches in clinical settings.

In summary, accumulating evidence highlights the pivotal role of the intratumoral microbiota in shaping immunotherapy responses in breast cancer. Through modulation of the tumor immune microenvironment, regulation of immune cell function, and interaction with inflammatory and metabolic pathways, these microbial communities exert multifaceted influences on treatment efficacy. However, significant gaps remain in understanding the dynamic interactions between microbiota and host immunity, as well as the causal relationships underlying these associations. In particular, the field still faces critical challenges including inter-patient variability, lack of standardized methodologies for low-biomass microbiome analysis, and insufficient longitudinal and mechanistic validation. The safety, controllability, and long-term effects of engineered bacteria and microbiome-based interventions also require careful evaluation before clinical translation. Furthermore, distinguishing causal microbial drivers from correlative signatures remains a key obstacle.

Inter-patient heterogeneity in microbial composition further underscores its potential relevance for precision medicine, suggesting that individualized microbiome profiling may be necessary to guide therapeutic decision-making. Future studies integrating multi-omics approaches, spatial resolution technologies, and well-designed clinical cohorts will be essential to elucidate these mechanisms. In addition, combining microbiome-based strategies with existing therapies, such as immune checkpoint inhibitors and targeted therapies, may offer synergistic benefits. Ultimately, translating microbiota-based insights into clinically actionable interventions will require coordinated efforts across basic research, translational studies, and clinical trials to fully harness the therapeutic potential of the intratumoral microbiome in breast cancer.

## 10 Intratumoral Microbiota in Chemotherapy Response and Resistance

### 10.1 Microbiota-Mediated Mechanisms of Chemotherapy and Targeted Therapy Resistance

Chemotherapy remains a cornerstone of comprehensive treatment for BC, particularly in patients with advanced or metastatic disease, and its efficacy is directly associated with patient survival outcomes [64,65]. However, the widespread development of chemoresistance severely compromises therapeutic efficacy, representing a major bottleneck in clinical management. In recent years, the regulatory role of the TME has emerged as a research focus, and intratumoral microbial communities, as critical components of the microenvironment, have been confirmed to directly modulate chemoresistance through multiple molecular mechanisms [66,67].

Mechanistically, the presence of intratumoral bacterial species, such as enterotoxigenic ETBF, can directly contribute to chemotherapy resistance in BC. ETBF, which is enriched in non-responders, encodes the bacterial toxin BFT-1, which specifically binds its functional receptor, NOD1, on the surface of tumor cells. The binding event activates the downstream signaling pathway that stimulates the phosphorylation and degradation of the tumor suppressor protein NUMB by cyclin G-associated kinase, thereby relieving the inhibition of the NOTCH1-HEY1 signaling pathway that regulates the enrichment or self-renewal of BC stem cells (BCSC), which are a hallmark subtype that shows inherent resistance to chemotherapeutic drugs such as docetaxel [22]. In targeted therapy, the intratumoral bacterium *P. aeruginosa* secretes the quorum-sensing molecule 3OC12-HSL (3OC), which directly facilitates ligand-independent dimerisation of the TGF-β type II receptor (TBRII) on the surface of cancer cells. This results in activation of the TGF-β pathway, ultimately leading to phosphorylation of ErbB2 and downstream activation of the MAPK and PI3K/Akt pathways, thereby contributing to resistance to the anti-ErbB2 monoclonal antibody trastuzumab [68]. Clinical studies have shown that higher levels of *P. aeruginosa* in the tumor are associated with resistance to trastuzumab therapy.

### 10.2 Intratumoral Microbiota as Predictive Biomarkers and Imaging Correlates of Treatment Response

While it has been directly shown that specific pathogens can promote chemoresistance, it has been discovered that the total number and diversity of intratumoral microbial communities serve as a non-invasive, highly predictive biomarker for successful treatment with combination therapies. In patients with TNBC who receive neoadjuvant chemoimmunotherapy (Chemo-IM), a greater intratumoral bacterial burden, as determined by 16S rDNA analysis and confirmed by fluorescence *in situ* hybridization and lipopolysaccharides, strongly predicts pCR. This prediction is specific to Chemo-IM and cannot be demonstrated with chemotherapeutic regimens per se. The bacterial signature indicative of improved outcomes has been shown to predict an immunologically active tumor environment, as evidenced by increased intratumoral anti-tumor CD4^+^CXCL13^+^ T cells and reduced numbers of intratumoral protumorigenic CD68^+^SPP1^+^ macrophages. Classifier models that include intratumoral microbial analysis outperform conventional clinicopathological models by a wide margin, thereby demonstrating potential as a tool for patient stratification [69].

Beyond serving as a direct biological target or systemic biomarker, the intratumoral microbiome can be non-invasively characterised and quantified through its imaging signatures. Radiomics and deep learning features extracted from pre- and mid-treatment MRI images are strongly correlated with intratumoral microbial density. Tumors with higher microbial density tend to display a concentric shrinking pattern after neoadjuvant therapy, making them more amenable to breast-conserving surgery. A multimodal fusion model integrating these microbiome-associated imaging features outperforms traditional imaging models in accurately predicting treatment response patterns, confirming the intratumoral microbiome as a core foundation for constructing non-invasive predictive models in BC [70].

Microbial-driven modulation of oncogenic signaling pathways and cancer stem cell dynamics highlights a direct mechanistic contribution to chemoresistance, while the association between microbial burden and treatment outcomes suggests important clinical utility for patient stratification. Nevertheless, the causal relationships between specific microbial taxa and therapeutic efficacy remain incompletely defined, and the heterogeneity of microbial composition across patients poses additional challenges for clinical translation. Future studies integrating longitudinal microbiome profiling with functional validation will be essential to clarify these interactions and to explore microbiota-targeted strategies aimed at overcoming resistance and improving therapeutic outcomes in BC.

## 11 Conclusion

Current evidence supports the presence of a reproducible intratumoral microbiome in BC, with distinct compositional patterns and associations with tumor subtype, clinicopathological features, and treatment response. Beyond descriptive profiling, accumulating experimental data suggest that tumor-associated microbes can actively modulate immune signaling, metabolic pathways, metastatic behavior, and therapeutic sensitivity, indicating that the intratumoral microbiome constitutes a functional component of the breast TME.

Nevertheless, most available data remain associative and are limited by low-biomass profiling challenges, methodological heterogeneity, and a lack of longitudinal and mechanistic validation. Future studies integrating standardized analytical pipelines, spatially resolved profiling, and functional models will be essential to clarify causality and determine whether intratumoral microbiome features can be reliably leveraged for clinical stratification or therapeutic intervention in BC. In the future, microbial signatures hold promise as predictive biomarkers for treatment selection, while targeting the intratumoral microbiome offers new therapeutic opportunities. Realizing these applications will require standardized detection protocols and prospective clinical validation.

## Data Availability

Not applicable. This article is a review and does not involve the generation or analysis of new experimental data.

## Supplementary Materials

The supplementary material is available online at https://www.techscience.com/doi/10.32604/or.2026.079281/s1.
