# Supplementary material for: The Intratumoral Microbiota in Breast Cancer: Roles in Progression, Immunity, and Therapy
Source: Oncol Res. 2026 Jul 16;34(8):9. doi: 10.32604/or.2026.079281 (PMC13397367; doi:10.32604/or.2026.079281)
Supplement: Supplementary file 1 [file OncolRes-34-79281-s001.zip › TSP_OR_79281-s001.docx]

**Supplementary Table S1:** Summary of key studies characterizing the intratumoral microbiome in breast cancer.

| **Study** | **Sample Types & Cohort Size** | **Detection Methods** | **Enriched Taxa** |
| --- | --- | --- | --- |
| **Urbaniak et al., 2014** | Ion Torrent V6 16S rRNA sequencing | Breast tissue | Higher relative abundance of proteobacteria |
| **Hieken et al., 2016** | Aseptically obtained human breast tissue from patients with benign breast disease (n=13) and invasive breast cancer (n=15). | 16S rRNA sequencing (Illumina MiSeq, V3-V5 region) | Higher relative abundance of bacteroidetes and firmicutes |
| **Urbaniak et al., 2016** | Breast tissue from healthy women, patients with benign tumors, and cancerous tumors (including normal adjacent tissues). | 16S rRNA gene sequencing (centered log-ratio transformed data). | **Cancerous Tissue:** Higher relative abundance of Bacillus, Staphylococcus, Enterobacteriaceae, Comamonadaceae, and Bacteroidetes. Healthy Tissue: Enriched in Prevotella, Lactococcus, Streptococcus, and Corynebacterium. |
| **Smith et al., 2019** | Normal breast (n=8), normal adjacent to tumor (n=11), and breast tumors (n=64) from non-Hispanic Black (NHB) and White (NHW) women. | 16S rRNA gene sequencing. | **Tumor Tissue:** Most abundant in Clostridia, Bacteroidia, and Ruminococcaceae.  **Racial/Subtype Differences:** Ralstonia (NHB), Xanthomonadaceae (NHW), and Streptococcaceae (Triple-Negative Breast Cancer). **Normal Tissue:** Higher abundance of Actinomycetaceae. |
| **Esposito et al., 2022** | Tumor breast tissues and paired adjacent non-tumoral tissue from women affected by breast cancer (n=34). | NGS-based 16S rRNA sequencing (V4-V6 regions). | **Tumor Tissue:** Firmicutes and Alphaproteobacteria were significantly overrepresented; exhibited lower overall richness. **Healthy/Adjacent Tissue:** Higher abundance of Actinobacteria (specifically Propionibacterium and Propionibacterium acnes). |
| **German et al., 2023** | Normal breast tissue cores from cancer-free women (n=403); tumor and/or adjacent normal tissue from BC patients (n=76). | 16S rRNA gene sequencing covering nine hypervariable regions (V1-V9). | **Tumor & Adjacent Tissue:** More abundant in Ralstonia (Proteobacteria phylum). **Normal Tissue:** Enriched in Lactobacillaceae, Acetobacterraceae (Acetobacter aceti), and Xanthomonadaceae (Xanthomonas sp.). |
| **Chang et al., 2025** | Breast cancer tissues and paired normal-appearing adjacent tissue (NAT) from clinical patients (n=13). | 16S rDNA sequencing (V3-V4 regions), | **Tumor Tissue:** Fusobacterium nucleatum is more abundant in breast cancer tissue than NAT |
| **Meng et al., 2018** | Aseptically collected breast tissue samples using percutaneous needle biopsy from patients with benign (n=22) and malignant (n=72) breast tumors. | 16S rRNA gene amplicon sequencing (V1-V2 regions) and PICRUSt. | **Malignant Tissue:** Enriched in Propionicimonas, Micrococcaceae, Caulobacteraceae, Rhodobacteraceae, Nocardioidaceae, and Methylobacteriaceae.  **Histological Grades:** With increasing malignancy (Grade I to III), the relative abundance of Bacteroidaceae decreased while Agrococcus increased. |
| **Urbaniak et al., 2014** | Breast tissue from 81 women (43 from Canada and 38 from Ireland; including cancerous, benign, and healthy reduction tissues). | 16S rRNA sequencing (V6 region) and bacterial culture. | Proteobacteria was the principal phylum, and the presence of viable bacteria was confirmed via culture.  **Geographical Discrepancies:** Canadian samples were enriched in Bacillus, Acinetobacter, Enterobacteriaceae, Pseudomonas, and Staphylococcus. Irish samples were enriched in Enterobacteriaceae, Staphylococcus, Listeria welshimeri, and Propionibacterium. |
| **Tzeng et al., 2021** | Fresh-frozen breast tissue from patients with breast cancer (n=221), individuals predisposed/high-risk (n=18), and healthy controls (n=69). | 16S rRNA gene sequencing (V3-V4 and V7-V9 regions) | **Tumor Tissue:** Decreased overall alpha-diversity. Anaerococcus, Caulobacter, and Streptococcus were major hubs in benign tissue but absent from cancer-associated tissues. |
| **Banerjee et al., 2021** | FFPE samples representing four breast cancer subtypes, matched controls, and non-matched controls from breast reductions. | Pan-pathogen microarray (PathoChip) targeting DNA and RNA | **Subtype Discrepancies:** ER+ tumors exhibited the most diverse microbiome, while Triple-Negative (TN) tumors were the least diverse. Signatures of Aggregatibacter were notably higher in TN. |
| **Mai et al., 2025** | Cancerous (n=20) and paracancerous tissues (n=21) from BC patients; 4T1 tumor-bearing mouse models. | 16S rDNA gene sequencing (V3-V4) | **Tumor Tissue:** Sphingobacterium multivorum displayed high abundance in cancerous tissues. |
